# Efficacy and Time-Dependent Pattern of Consolidation Immunotherapy in Stage III Non-Small Cell Lung Cancer After Induction Chemoimmunotherapy and Radiotherapy: A Dual-Center Retrospective Cohort Study

**DOI:** 10.3390/cancers18132035

**Published:** 2026-06-23

**Authors:** Hao Zhang, Yujun Hu, Ciming Sun, Huimin Xu, Yajing Liang, Hui Liu, Qiwen Li, Shuohan Zheng

**Affiliations:** 1 Department of Radiation Oncology, Hubei Cancer Hospital, Tongji Medical College, Huazhong University of Science and Technology, Wuhan 430079, China; zhanghao52002@163.com; 2 Department of Radiology, State Key Laboratory of Oncology in South China, Guangdong Key Laboratory of Nasopharyngeal Carcinoma Diagnosis and Therapy, Guangdong Provincial Clinical Research Center for Cancer, Sun Yat-sen University Cancer Center, Guangzhou 510060, China; huyj1@sysucc.org.cn; 3 Department of Radiation Oncology, State Key Laboratory of Oncology in South China, Guangdong Key Laboratory of Nasopharyngeal Carcinoma Diagnosis and Therapy, Guangdong Provincial Clinical Research Center for Cancer, Sun Yat-sen University Cancer Center, Guangzhou 510060, China; suncm@sysucc.org.cn (C.S.); liangyaj@sysucc.org.cn (Y.L.); liuhui4@sysucc.org.cn (H.L.); 4 United Laboratory of Frontier Radiotherapy Technology of Sun Yat-sen University & Chinese Academy of Sciences Ion Medical Technology Co., Ltd., Guangzhou 510060, China; 5 Department of Medical Oncology, Yiyang Center Hospital, Yiyang 413000, China; xuhuimin0203@163.com

**Keywords:** consolidation immunotherapy, unresectable stage III non-small cell lung cancer, progression-free survival, overall survival

## Abstract

Background/Objectives: The efficacy and time-dependent pattern of consolidation immunotherapy after induction chemoimmunotherapy and radiotherapy remain unclear in unresectable stage III non-small cell lung cancer (NSCLC). This study evaluated the efficacy and temporal pattern of consolidation immunotherapy in this population. Methods: This dual-center retrospective cohort study included patients with unresectable stage III NSCLC who achieved disease control after induction chemoimmunotherapy followed by definitive radiotherapy. To reduce potential immortal time bias, only patients who remained alive and progression-free within 2 months after radiotherapy were included. Patients were grouped according to receipt of consolidation immunotherapy. Survival outcomes were analyzed using stabilized inverse probability of treatment weighting, multivariable Cox regression, sequential landmark Cox analysis, and landmark restricted mean survival time analysis. Results: Among 170 eligible patients, 65 received consolidation immunotherapy and 105 did not. After a median follow-up of 33 months, consolidation immunotherapy was associated with longer PFS (hazard ratio [HR]: 0.52, 95% confidence interval [CI]: 0.35–0.78, *p* = 0.001) and OS (HR: 0.35, 95% CI: 0.18–0.65, *p* < 0.001). These benefits remained significant after weighting, and multivariable analysis identified consolidation immunotherapy as an independent predictor of improved PFS. Exploratory sequential landmark Cox and restricted mean survival time analyses suggested that the survival benefit was more evident during the early treatment period, particularly around 8–10 months. Conclusions: Consolidation immunotherapy was associated with improved survival, and its benefit appeared more evident during the early treatment period, particularly around 8–10 months. The optimal treatment duration requires further prospective validation.

## Simple Summary

This dual-center retrospective cohort study assessed the efficacy and time-dependent pattern of consolidation immunotherapy in patients with unresectable stage III non-small cell lung cancer who achieved disease control after induction chemoimmunotherapy followed by radiotherapy. To reduce potential immortal time bias, only patients who remained alive and progression-free within 2 months after radiotherapy were included. Among 170 patients, 65 received consolidation immunotherapy and 105 did not. After a median follow-up of 33 months, consolidation immunotherapy was associated with improved progression-free survival (PFS) and overall survival. These benefits remained significant after stabilized inverse probability of treatment weighting, and multivariable analysis identified consolidation immunotherapy as an independent predictor of PFS. Exploratory sequential landmark Cox and restricted mean survival time analyses suggested that the survival benefit was more evident during the early treatment period, particularly around 8–10 months.

## 1. Introduction

Approximately one-third of patients with non-small cell lung cancer (NSCLC) present with locally advanced stage III disease [1,2]. This stage is highly heterogeneous, and a substantial proportion of patients have unresectable tumors at diagnosis [3,4]. Although definitive radiotherapy or chemoradiotherapy remains the cornerstone of curative-intent treatment for unresectable stage III NSCLC, long-term disease control remains challenging due to the persistent risks of locoregional recurrence and distant metastasis [5,6,7,8].

Immune checkpoint inhibitors have reshaped the treatment landscape for unresectable stage III NSCLC [9,10]. The PACIFIC trial established consolidation durvalumab after definitive chemoradiotherapy as the standard of care for patients without disease progression, with treatment administered for up to 12 months [6]. Long-term follow-up has confirmed persistent improvements in progression-free survival (PFS) and overall survival (OS) [11]. Moreover, the GEMSTONE-301 trial demonstrated that consolidation sugemalimab improved PFS after either concurrent or sequential chemoradiotherapy [12], suggesting that the benefit of immune consolidation may extend beyond the conventional concurrent chemoradiotherapy setting.

Against this background, induction chemotherapy combined with immunotherapy before definitive local treatment has emerged as a strategy of increasing interest for selected patients with unresectable stage III NSCLC. This approach may reduce tumor burden before radiotherapy, improve the feasibility and tolerability of subsequent radical local treatment, and provide earlier systemic control of micrometastatic disease [13,14]. From a biological perspective, induction immunotherapy may initiate systemic antitumor immune activation, whereas subsequent radiotherapy may enhance antigen release and immunogenic cell death, thereby supporting the combination of induction chemoimmunotherapy with definitive radiotherapy or chemoradiotherapy [15,16,17].

Several studies have explored this intensified multimodality strategy [13,14,18]. Wang et al. investigated induction of immune checkpoint inhibitors plus chemotherapy before definitive chemoradiotherapy in bulky unresectable stage III NSCLC. They reported a median PFS of 30.6 months, with tumor reduction and improved dosimetric feasibility for subsequent radiotherapy [13]. The phase II APOLO trial further investigated induction chemoimmunotherapy followed by chemoradiotherapy and maintenance immunotherapy in unresectable stage III NSCLC, demonstrating encouraging activity with manageable safety, supporting the feasibility of this treatment sequence [14]. These observations suggest that induction chemoimmunotherapy followed by definitive local therapy may be a promising strategy, although its role in routine clinical practice remains unclear.

Most previous studies have either evaluated consolidation immunotherapy after chemoradiotherapy or examined induction chemoimmunotherapy, chemoradiotherapy, and maintenance immunotherapy as an integrated treatment strategy. However, for patients who have already received induction chemoimmunotherapy and achieved disease control following definitive radiotherapy or chemoradiotherapy, it remains uncertain whether continued immunotherapy confers additional survival benefit. The optimal duration of continued immunotherapy in this setting also remains undefined.

Accordingly, we conducted a dual-center retrospective cohort study to evaluate the efficacy of consolidation immunotherapy in patients with unresectable stage III NSCLC who achieved disease control after induction chemoimmunotherapy followed by radiotherapy. Specifically, we aimed to determine whether continued immunotherapy provides survival benefits and to characterize its time-dependent treatment effects in this population.

## 2. Materials and Methods

### 2.1. Study Design and Patient Selection

This retrospective cohort study included patients with unresectable stage III NSCLC treated at Sun Yat-sen University Cancer Center and Hubei Cancer Hospital between August 2018 and December 2023. Eligibility criteria included: (1) histologically confirmed NSCLC; (2) unresectable stage III disease according to the eighth edition of the American Joint Committee on Cancer (AJCC) staging system; (3) induction chemotherapy plus immunotherapy followed by definitive radiotherapy or chemoradiotherapy; (4) disease control after radiotherapy, defined as complete response, partial response, or stable disease according to Response Evaluation Criteria in Solid Tumors (RECIST) 1.1; and (5) complete clinical and follow-up data for survival analysis. Patients were excluded if they had undergone surgical resection or local ablative therapy, harbored EGFR mutations or ALK rearrangements, did not receive radiotherapy, did not receive induction immunotherapy, or had a concurrent or prior history of another malignancy.

To minimize potential immortal time bias arising from differences in post-radiotherapy eligibility and the timing of consolidation immunotherapy initiation, we applied a uniform 2-month post-radiotherapy landmark eligibility window. Specifically, only patients who remained alive and free of disease progression within 2 months after completion of radiotherapy were included. Patients who initiated consolidation immunotherapy within this window were assigned to the consolidation immunotherapy group; those who remained progression-free but did not receive consolidation immunotherapy were assigned to the non-consolidation immunotherapy group.

### 2.2. Treatment

All patients received induction chemotherapy with platinum-based doublet regimens, including taxane, pemetrexed for non-squamous, or etoposide plus platinum administered every 3 weeks for at least one cycle. Definitive thoracic radiotherapy was delivered using intensity-modulated radiotherapy or volumetric-modulated arc therapy, with a median prescribed dose of 60.0 Gy (interquartile range [IQR]: 60.0–63.9 Gy). Concurrent chemotherapy was administered at the physician’s discretion. Consolidation immunotherapy with anti-PD-1 or anti-PD-L1 agents were administered every 3–4 weeks according to standard dosing schedules until disease progression, unacceptable toxicity, or completion of planned treatment duration.

### 2.3. Response Assessment and Follow-Up

Tumor response was assessed according to RECIST, version 1.1. Chest computed tomography (CT) was used to evaluate primary lesions, whereas CT, magnetic resonance imaging, emission computed tomography (ECT), and positron emission tomography/CT were used to evaluate distant metastases. Radiologic assessments were performed every 2–3 treatment cycles and every 3 months during follow-up. The primary endpoint was PFS, defined for the primary efficacy analysis as the time from initiation of chemotherapy to disease progression or death from any cause. OS was assessed as the secondary endpoint and was defined as the time from initiation of chemotherapy to death from any cause or to the date of last follow-up. For analyses examining the time-dependent benefit of consolidation immunotherapy, landmark-PFS was defined as the time from radiotherapy completion to disease progression or death and was used in the sequential landmark Cox analysis and landmark-restricted mean survival time (RMST). Adverse events (AEs) were documented and graded according to the Common Terminology Criteria for Adverse Events (CTCAE), version 5.0.

### 2.4. Statistical Analysis

Continuous variables are presented as medians with IQRs, and categorical variables as frequencies and percentages. Groups were compared using the Mann–Whitney U-test, chi-square test, or Fisher’s exact test, as appropriate. Survival curves were estimated using the Kaplan–Meier method and compared using the log-rank test. Hazard ratios (HRs) and 95% confidence intervals (CIs) were estimated with Cox proportional hazards regression models.

To reduce baseline differences and confounding between the consolidation and non-consolidation immunotherapy groups, inverse probability of treatment weighting (IPTW) based on propensity scores was applied [19,20]. Propensity scores were estimated using a multivariable logistic regression model with consolidation immunotherapy as the dependent variable. Covariates included age, sex, smoking status, histology, disease stage, induction chemotherapy regimen, concurrent chemotherapy, and radiotherapy dose. Stabilized inverse probability weights were calculated to reduce the influence of extreme weights and improve the stability of the estimates. For patients who received consolidation immunotherapy, the stabilized weight was the marginal probability of receiving consolidation immunotherapy divided by the individual propensity score; for those who did not, it was defined as the marginal probability of not receiving consolidation immunotherapy divided by one minus the individual propensity score. After weighting, covariate balance was assessed using standardized mean differences (SMDs), with an absolute value < 0.1 indicating adequate balance.

To characterize the time-dependent association between consolidation immunotherapy and disease progression, we performed sequential landmark Cox regression analysis [21,22]. At each prespecified landmark time point, only patients who survived and were progression-free were included in the risk set. Follow-up was recalculated from the landmark, and Cox proportional hazards models were used to estimate HR for subsequent disease progression between consolidation and non-consolidation immunotherapy groups. For example, at the 6-month landmark, only patients who remained progression-free during the first six months were included, and the risk of progression thereafter was compared between groups. HRs across serial landmark time points were plotted to illustrate the temporal pattern of treatment benefits.

Landmark-RMST analysis was used to quantify the cumulative survival benefit of maintenance immunotherapy across treatment durations [23,24,25]. Prespecified landmark time points ranged from 2 to 22 months, and RMST was evaluated at truncation times of 18, 24, 30, and 36 months. For each landmark analysis, patients with disease progression or who died before the landmark were excluded, and survival time was recalculated from that point. Differences in RMST were calculated as the value in the consolidation immunotherapy group minus the value in the control group. Only valid combinations with τ greater than the landmark time were analyzed. A positive ΔRMST indicated longer restricted mean PFS with maintenance immunotherapy.

All statistical analyses were performed using the R software (version 4.4.3; R Foundation for Statistical Computing, Vienna, Austria). A two-sided *p*-value < 0.05 was considered statistically significant.

## 3. Results

### 3.1. Patient Characteristics

**Figure 1:**
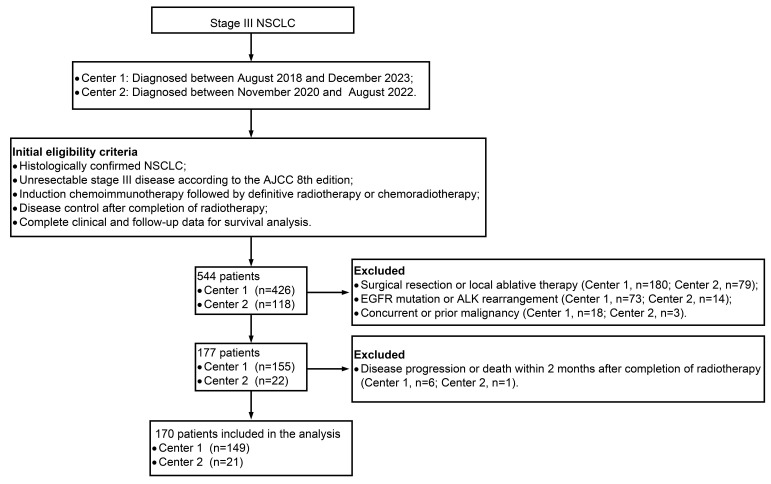
Patient enrollment flowchart. Center 1: Sun Yat-sen University Cancer Center; Center 2: Hubei Cancer Hospital.

A total of 170 patients with unresectable stage III NSCLC were included, of whom 149 were treated at Sun Yat-sen University Cancer Center and 21 at Hubei Cancer Hospital. The patient enrollment flowchart is shown in Figure 1. All patients achieved disease control after induction chemoimmunotherapy followed by radiotherapy. Among them, 65 received consolidation immunotherapy, while 105 patients did not. In the consolidation immunotherapy group, the median duration of consolidation immunotherapy was 8 months (IQR, 3–14 months). Baseline characteristics before and after IPTW adjustment are summarized in Table 1 and Table S1.

Before IPTW adjustment, several variables, including histology, disease stage, induction chemotherapy regimen, and radiotherapy dose, were imbalanced, with SMDs greater than 0.2. After stabilized IPTW adjustment, covariate balance improved substantially, with most SMDs falling below 0.1. A small residual imbalance remained for the induction chemotherapy regimen, with the SMD decreasing from 0.32 before IPTW to 0.11 after IPTW adjustment (Table 1). The stabilized weight distribution was also acceptable, with a mean of 0.99, a median of 0.94, a maximum of 3.6, and 1st and 99th percentiles of 0.50 and 2.24, respectively (Figure S1). These findings indicated adequate overlap between treatment groups and no evidence that extreme weights drove the weighted estimates; therefore, weight truncation was not performed.

### 3.2. Efficacy of Consolidation Immunotherapy for NSCLC

**Figure 2:**
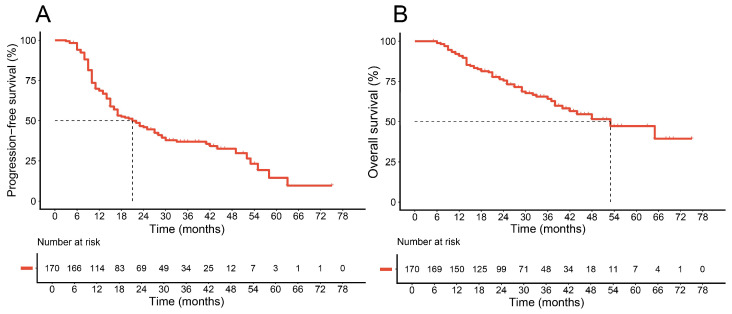
Kaplan–Meier survival curves for the overall cohort. (**A**) Progression-free survival and (**B**) overall survival in patients with unresectable stage III NSCLC who achieved disease control after induction chemoimmunotherapy followed by radiotherapy. The red solid line represents the Kaplan–Meier survival curve.The dashed horizontal line indicates 50% survival probability, and the dashed vertical line indicates the corresponding median survival time. Abbreviations: NSCLC, Non-Small Cell Lung Cancer.

**Figure 3:**
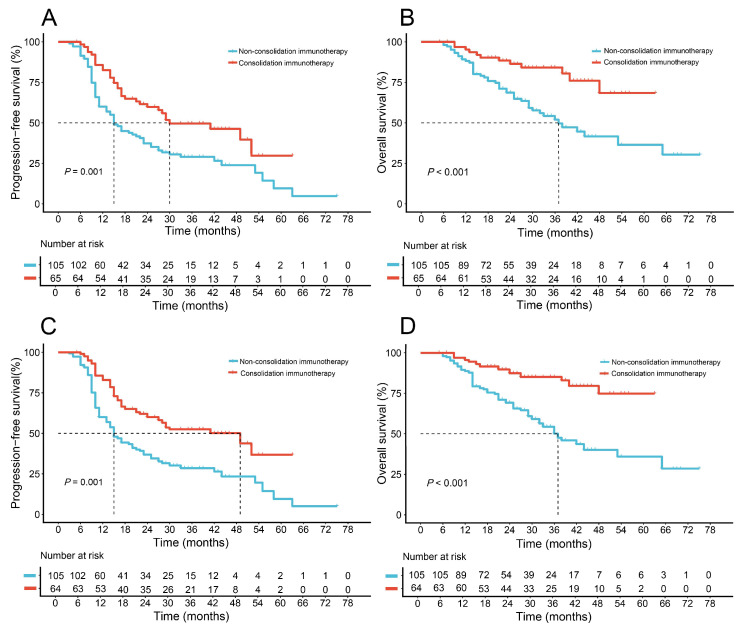
Kaplan–Meier curves comparing survival outcomes between the consolidation immunotherapy and non-consolidation immunotherapy groups before and after IPTW adjustment. (**A**) Progression-free survival before IPTW adjustment. (**B**) Overall survival before IPTW adjustment. (**C**) Progression-free survival after IPTW adjustment. (**D**) Overall survival after IPTW adjustment. The dashed horizontal line indicates 50% survival probability, and the dashed vertical line indicates the corresponding median survival time. Abbreviations: IPTW, inverse probability of treatment weighting; NSCLC, non-small cell lung cancer; PFS, progression-free survival; OS, overall survival.

The median follow-up for the overall cohort was 33 months (95% CI, 31–38 months). The median PFS and OS were 21 months and 53 months, respectively (Figure 2A,B). In the unadjusted cohort, consolidation immunotherapy was associated with significantly improved survival. Median PFS was 30 months in the consolidation immunotherapy group versus 15 months in the non-consolidation immunotherapy group (HR: 0.52, 95% CI: 0.35–0.78, *p* = 0.001; Figure 3A). Similarly, median OS was longer in the consolidation immunotherapy group (not reached vs. 37 months; HR: 0.35, 95% CI: 0.18–0.65, *p* < 0.001; Figure 3B). After IPTW adjustment, these associations remained robust. Weighted Kaplan–Meier analysis showed significantly longer PFS (HR: 0.47, 95% CI: 0.30–0.75, *p* = 0.001; Figure 3C) and OS (HR: 0.29, 95% CI: 0.15–0.57, *p* < 0.001; Figure 3D) in the consolidation immunotherapy group. These findings indicate a consistent survival advantage associated with consolidation immunotherapy independent of baseline imbalances.

In multivariable analysis, consolidation immunotherapy remained an independent predictor of improved PFS (HR: 0.52, 95% CI: 0.34–0.80, *p* = 0.002; Table 2). To further address potential residual imbalance in treatment-related variables, we performed a weighted multivariable Cox sensitivity analysis adjusting for induction chemotherapy regimen and other treatment-related factors. The association between consolidation immunotherapy and improved PFS was unchanged in this sensitivity analysis (HR: 0.49, 95% CI: 0.31–0.76, *p* = 0.001; Table 2), supporting the robustness of the findings.

In subgroup analyses stratified by concurrent chemotherapy status, consolidation immunotherapy was associated with improved PFS both in patients who received concurrent chemotherapy and in those who did not. This PFS benefit remained statistically significant in the concurrent chemotherapy subgroup (HR: 0.55, 95% CI: 0.34–0.89, *p* = 0.01) and in the subgroup without concurrent chemotherapy (HR: 0.47, 95% CI: 0.23–0.96, *p* = 0.04), suggesting that the benefit of consolidation immunotherapy was generally consistent regardless of concurrent chemotherapy use (Figure S2).

### 3.3. Time-Dependent Pattern of Consolidation Immunotherapy for NSCLC

**Figure 4:**
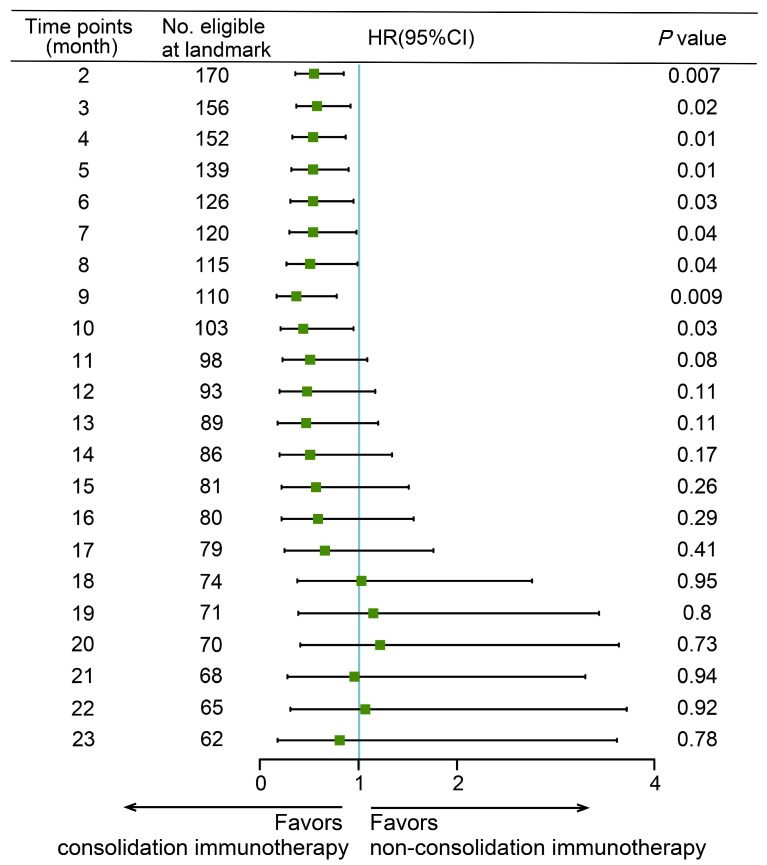
Sequential landmark Cox analysis of consolidation immunotherapy over time. Hazard ratios for subsequent disease progression were estimated using Cox proportional hazards models at sequential landmark time points among patients who remained progression-free at each landmark. The vertical reference line indicates HR = 1. HRs less than 1 favored consolidation immunotherapy. Abbreviations: HR, hazard ratio; CI, confidence interval.

Sequential landmark Cox proportional hazards models were used to assess the time-dependent effect of consolidation immunotherapy on subsequent disease progression among patients who remained alive and progression-free at each landmark. Consolidation immunotherapy was associated with a significantly lower risk of progression during early follow-up (Figure 4). From months 2 to 10, HRs consistently favored the consolidation immunotherapy group, ranging from 0.37 to 0.58 (all *p* < 0.05). The greatest benefit was observed at approximately month 9 (HR: 0.37, 95% CI: 0.17–0.78, *p* = 0.009). Thereafter, the magnitude of benefit gradually decreased. After 11 months, the difference between groups was no longer statistically significant, with HRs approaching 1.0 (all *p* > 0.05). Beyond 17 months, no apparent survival advantage was observed, and HRs fluctuated around unity. Overall, these findings suggest a time-dependent survival benefit of consolidation immunotherapy, with the greatest effect observed within the first 10 months and attenuation beginning at approximately month 11.

**Figure 5:**
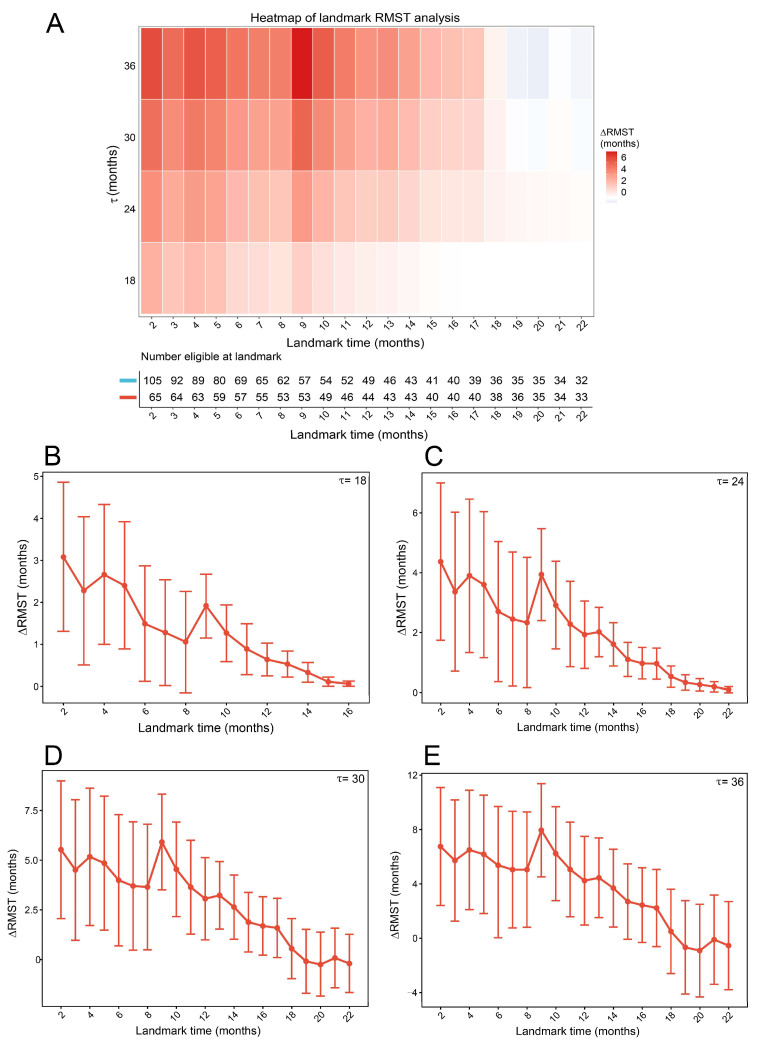
Landmark RMST analysis of consolidation immunotherapy duration. (**A**) Heatmap of ΔRMST between the consolidation immunotherapy and non-consolidation immunotherapy groups across serial landmark times and truncation times. In the eligibility table, blue represents the non-consolidation immunotherapy group, and red represents the consolidation immunotherapy group. (**B**–**E**) Line plots of ΔRMST at truncation times of τ = 18, 24, 30, and 36 months, respectively. Positive ΔRMST values favor consolidation immunotherapy. Abbreviations: RMST, restricted mean survival time.

Landmark RMST analysis was performed to further explore the optimal duration of consolidation immunotherapy. The heatmap and line plots demonstrated a consistent pattern of time-dependent treatment benefit. Across most τ settings, the greatest increase in ΔRMST was observed at earlier landmark time points, with the maximal benefit occurring at approximately 8–10 months, except for the τ = 18-month analysis (Figure 5A). As the landmark time increased, ΔRMST gradually decreased. For τ = 18 months, the benefit declined rapidly after approximately 10–12 months and approached zero thereafter (Figure 5B). Similarly, for τ = 24 months, ΔRMST remained positive for approximately 14–17 months but diminished progressively with longer landmark times (Figure 5C). For longer truncation times (τ = 30 and 36 months; Figure 5D,E), the decline in ΔRMST was more gradual; however, the survival advantage was no longer evident beyond approximately 18 months, with some estimates approaching or crossing zero.

Taken together, the sequential landmark Cox analysis and landmark RMST analyses consistently suggested that the benefit of consolidation immunotherapy was most pronounced during the early treatment period, particularly within approximately the first 8–10 months.

### 3.4. Safety Analysis

Adverse events were further compared between the consolidation immunotherapy and non-consolidation immunotherapy groups. Overall, the safety profiles were comparable between the two groups, with no statistically significant differences in the incidence of any-grade or grade 3–4 adverse events. The most common adverse events in both groups were anemia, pneumonia, hyperglycemia, hypercholesterolemia, and myelosuppression. Although no statistically significant differences were observed, the consolidation immunotherapy group showed numerically higher incidences of pneumonia, myelosuppression, pyrexia, hypothyroidism, blood creatinine increase, hypercholesterolemia, hyperglycemia, and hypercalcemia than the non-consolidation immunotherapy group. Overall, the incidence of grade 3–4 adverse events was relatively low in both groups. The incidence of grade 3–4 myelosuppression was slightly higher in the consolidation immunotherapy group than in the non-consolidation immunotherapy group. In contrast, the incidences of grade 3–4 anemia, pneumonia, hyperglycemia, hypothyroidism, and elevated liver enzymes were numerically higher in the non-consolidation immunotherapy group, although no statistically significant differences were observed between groups. One grade 5 pneumonia event occurred in the non-consolidation immunotherapy group, whereas no grade 5 adverse events were observed in the consolidation immunotherapy group.

After excluding patients who experienced disease progression within 2 months after completion of radiotherapy, we further reviewed the documented reasons for not receiving consolidation immunotherapy in the non-consolidation immunotherapy group and for receiving consolidation immunotherapy for less than 2 years in the consolidation immunotherapy group. In the non-consolidation immunotherapy group, the documented reasons for not receiving consolidation immunotherapy included pneumonia of varying grades in 8 patients (7.6%), personal reasons in 2 patients (1.9%), and the COVID-19 pandemic in 2 patients (1.9%); no specific reason was documented in the remaining patients (88.6%). In the consolidation immunotherapy group, 58 patients did not complete 2 years of consolidation immunotherapy. The documented reasons included disease progression in 17 patients (29.3%), pneumonia of varying grades in 6 patients (10.3%), the COVID-19 pandemic in 2 patients (3.4%), other comorbidities in 3 patients (5.2%), personal reasons in 1 patient (1.7%), and subdural hemorrhage unrelated to cancer treatment in 1 patient (1.7%). The reason was not documented in the remaining 28 patients (48.4%).

## 4. Discussion

In this dual-center retrospective cohort study, consolidation immunotherapy was associated with significantly prolonged PFS and OS in patients with unresectable stage III NSCLC who achieved disease control after induction chemoimmunotherapy and radiotherapy. These associations remained robust after IPTW adjustment and multivariable analysis. Exploratory sequential landmark Cox analysis and landmark RMST analyses further suggested time-dependent pattern of benefit, with the greatest effect observed during the first 8–10 months.

Consolidation immunotherapy has emerged as a central component of multimodal cancer treatment for unresectable stage III NSCLC. To date, however, the supporting evidence has been derived predominantly from cohorts treated after concurrent or sequential chemoradiotherapy [6,12,26]. In the PACIFIC trial, durvalumab administered after definitive chemoradiotherapy conferred durable improvements in PFS and OS, establishing 12 months of consolidation durvalumab as the standard of care [6]. Four-year follow-up confirmed sustained long-term benefits, with superior OS and PFS rates relative to the placebo group [11]. Real-world data from PACIFIC-R further supported the feasibility of this approach, with a median durvalumab exposure of 337 days [27]. Likewise, GEMSTONE-301 demonstrated that sugemalimab improved PFS after either concurrent or sequential chemoradiotherapy in patients with unresectable stage III NSCLC, extending the rationale for immune consolidation beyond the classic concurrent chemoradiotherapy paradigm [12]. PACIFIC-6 further supported the safety and activity of durvalumab after sequential chemoradiotherapy in patients considered unsuitable for concurrent chemoradiotherapy in real-world practice [26].

The survival advantage observed in the current study is consistent with the established role of consolidation immunotherapy in unresectable stage III NSCLC. However, our cohort differed from those of prior studies in that all patients had achieved disease control after induction chemoimmunotherapy, followed by radiotherapy. This sequence may have selected patients with more favorable tumor biology and greater treatment sensitivity, thereby contributing in part to the favorable outcomes observed. More broadly, these findings extend the current evidence base by suggesting that continued immunotherapy may retain clinical value even after disease control has already been achieved through induction chemoimmunotherapy and radiotherapy.

The optimal duration of consolidation immunotherapy in stage III NSCLC remains unresolved, particularly in patients treated with induction chemoimmunotherapy before radiotherapy. Although the PACIFIC regimen specifies durvalumab for up to 12 months [6], treatment duration in other studies and in real-world practice has been variable. The available evidence suggests that immunotherapy should be maintained for a sufficient period to achieve durable benefits, yet prolonged or indefinite administration may not yield proportional gains. In CheckMate 153, discontinuing nivolumab after 1 year appeared to be insufficient for some patients with advanced NSCLC [28]. By contrast, real-world data from Sun et al. suggested that, among patients who remained progression-free after 2 years of immunotherapy, indefinite continuation of treatment was not associated with improved OS compared with discontinuation at 2 years [29]. Comparable observations in recurrent or metastatic head and neck squamous cell carcinoma further support the concept that treatment beyond a fixed duration may not invariably confer additional benefit [30,31]. Consistent with this, our previous study on de novo metastatic nasopharyngeal carcinoma suggested that approximately 15 months of consolidation immunotherapy may be sufficient after disease control [31].

In this study, sequential landmark Cox analysis and landmark RMST analyses provided complementary exploratory insight into the temporal dynamics of benefit from consolidation immunotherapy. Sequential landmark Cox analysis indicated that the relative treatment effect appeared more pronounced during the first 10 months and began to attenuate after month 11. In parallel, landmark RMST analysis suggested that the absolute survival gain was more evident around 8–10 months and diminished thereafter. These findings underscore the complementary strengths of the two approaches: sequential landmark Cox analysis reflects relative risk reduction from a given landmark onward, whereas RMST captures the cumulative absolute survival benefit over a defined time horizon. Considered together, these exploratory analyses suggest that the clinical benefit of consolidation immunotherapy may be more evident during the early treatment period, particularly around 8–10 months. Nonetheless, these observations should be interpreted as hypothesis-generating, as this study was not designed or powered to compare prespecified treatment durations.

This study should be interpreted in light of several limitations. First, the retrospective, nonrandomized design may have introduced selection bias and residual confounding. Although a uniform 2-month post-radiotherapy eligibility window, stabilized IPTW, and multivariable Cox models were used to reduce bias and balance measured covariates, including induction chemotherapy regimen, concurrent chemotherapy, radiotherapy dose, and radiotherapy technique, unmeasured factors such as physician preference, patient willingness, treatment tolerance, socioeconomic status, and drug accessibility may still have influenced treatment outcomes and limited the generalizability. In addition, PD-L1 expression and tumor mutational burden were not routinely assessed during the study period, and their potential influence on treatment outcomes could not be fully evaluated. Second, although this was a dual-center study, most patients were enrolled from one center, and potential center-related differences in treatment practice, follow-up strategy, or patient selection may have affected the results. Third, given the modest sample size, particularly at later landmark time points, the sequential landmark Cox analysis and landmark RMST estimates should be interpreted with appropriate caution. Fourth, adverse events were retrospectively extracted from medical records and may have been underreported, and the lack of detailed toxicity data may further limit the generalizability of the safety findings; therefore, the observed incidences should be interpreted with caution. Finally, this study did not directly compare predefined treatment durations. Accordingly, the observed time-dependent pattern of benefit should be considered exploratory and hypothesis-generating rather than definitive evidence regarding the optimal treatment duration.

## 5. Conclusions

This real-world study suggests that consolidation immunotherapy is associated with meaningful survival benefits in patients with unresectable stage III NSCLC who achieve disease control after induction chemoimmunotherapy and radiotherapy. Exploratory conditional survival and landmark RMST analyses further suggest that this benefit may be more evident during the early treatment period, particularly around 8–10 months. These findings provide a clinically relevant hypothesis regarding the time-dependent pattern of the benefit from consolidation immunotherapy, but they should not be interpreted as definitive evidence for an optimal treatment duration. Prospective studies are warranted to determine whether shorter, similar, or longer treatment courses can provide comparable outcomes.

## Figures and Tables

**Table 1:** Baseline characteristics of patients with unresectable stage III NSCLC according to consolidation immunotherapy before and after IPTW adjustment.

Variable	Overall Cohort				IPTW-Adjusted Cohort			
	Non-Consolidation Immunotherapy Group (*n* = 105)	Consolidation Immunotherapy Group (*n* = 65)	*p* Value	SMD	Non-Consolidation Immunotherapy Group (*n* = 105)	Consolidation Immunotherapy Group (*n* = 64)	*p* Value	SMD
Age, median (IQR) (years)	63 (57–67)	63 (57–66)	0.68	0.11	62 (57–67)	64 (57–66)	0.65	0.06
Sex			0.84	0.07			0.81	0.04
Male	96 (91.4)	58 (89.2)			96 (91.4)	59 (92.2)		
Female	9 (8.6)	7 (10.8)			9 (8.6)	5 (7.8)		
Smoking			0.54	0.14			0.96	0.007
No	10 (9.5)	9 (13.8)			11 (10.5)	7 (10.9)		
Yes	95 (90.5)	56 (86.2)			94 (89.5)	57 (89.1)		
Histology			0.25	0.27			0.98	0.03
Squamous cell carcinoma	59 (56.2)	40 (61.5)			61 (58.1)	38 (59.4)		
Adenocarcinoma	34 (32.4)	14 (21.5)			30 (28.6)	17 (26.6)		
Others	12 (11.4)	11 (16.9)			14 (13.3)	9 (14.0)		
Stage			0.20	0.28			0.92	0.06
IIIA	29 (27.6)	16 (24.6)			28 (26.7)	16 (25.0)		
IIIB	59 (56.2)	31 (47.7)			55 (52.3)	33 (51.6)		
IIIC	17 (16.2)	18 (27.7)			22 (21.0)	15 (23.4)		
Concurrent chemotherapy			0.29	0.19			0.94	0.01
No	42 (40.0)	20 (30.8)			38(36.2)	23 (35.9)		
Yes	63 (60.0)	45 (69.2)			67 (63.8)	41 (64.1)		
Induction chemotherapy regimen			0.28	0.32			0.93	0.11
TP	67 (63.8)	50 (76.9)			71 (67.6)	45 (70.4)		
AP	26 (24.8)	12 (18.5)			24 (22.9)	15 (23.4)		
EP	7 (6.7)	2 (3.1)			6 (5.7)	2 (3.1)		
Others	5 (4.8)	1 (1.5)			4 (3.8)	2 (3.1)		
Radiation dose Median (IQR), (Gy)			0.04	0.38			0.96	0.009
<60	24 (22.9)	6 (9.2)			19 (18.1)	11 (17.2)		
≥60	81 (77.1)	59 (90.8)			86 (81.9)	53 (82.8)		

Abbreviation: NSCLC, Non-Small Cell Lung Cancer; IPTW, Inverse Probability of Treatment Weighting; SMDs, Standardized Mean Differences.

**Table 2:** Multivariable Cox regression analysis for progression-free survival before and after IPTW adjustment.

Variable	Before IPTW Adjustment			After IPTW Adjustment		
	HR	95% CI	*p* Value	Weighted HR	95% CI	*p* Value
Age	0.99	0.96–1.02	0.55	0.99	0.96–1.02	0.55
Sex						
Male	Reference			Reference		
Female	0.90	0.44–1.84	0.78	1.15	0.58–2.30	0.69
Smoking						
No	Reference			Reference		
Yes	0.69	0.39–1.21	0.20	0.63	0.35–1.14	0.13
Stage						
IIIA	Reference			Reference		
IIIB	1.38	0.88–2.18	0.16	1.36	0.84–2.20	0.21
IIIC	0.96	0.52–1.80	0.90	0.98	0.51–1.90	0.95
Concurrent chemotherapy						
No	Reference			Reference		
Yes	1.05	0.69–1.58	0.83	1.05	0.68–1.62	0.82
Induction chemotherapy regimen						
TP	Reference					
AP	0.78	0.49–1.24	0.30	0.78	0.47–1.27	0.32
EP	1.30	0.56–2.99	0.54	1.17	0.52–2.63	0.71
Others	1.83	0.66–5.06	0.25	2.27	0.82–6.31	0.11
Radiation dose Median (IQR), (Gy)						
<60	Reference					
≥60	0.96	0.58–1.60	0.88	1.26	0.70–2.26	0.44
Consolidation immunotherapy						
No	Reference			Reference		
Yes	0.52	0.34–0.80	0.002	0.49	0.31–0.76	0.001

Abbreviation: IPTW, Inverse Probability of Treatment Weighting; HR, Hazard ratio; CI, Confidence interval.

## Data Availability

The raw data obtained during the present study was collected under institutional ethics approval. Data can be obtained from the corresponding author upon reasonable request and completion of the required approval process.

## Supplementary Materials

The following supporting information can be downloaded at https://www.mdpi.com/article/10.3390/cancers18132035/s1: Table S1: Additional baseline characteristics of patients according to consolidation immunotherapy. Table S2. Adverse events in consolidation immunotherapy and non-consolidation immunotherapy group. Figure S1. Distribution of stabilized inverse probability of treatment weighting (IPTW) weights. Figure S2. Kaplan–Meier curves of progression-free survival stratified by concurrent chemotherapy status.

## References

[1] Petrella F., Rizzo S., Attili I., Passaro A., Zilli T., Martucci F., Bonomo L., Del Grande F., Casiraghi M., De Marinis F. (2023). Stage III Non-Small-Cell Lung Cancer: An Overview of Treatment Options. Curr. Oncol..

[2] Miao D., Zhao J., Han Y., Zhou J., Li X., Zhang T., Li W., Xia Y. (2024). Management of locally advanced non-small cell lung cancer: State of the art and future directions. Cancer Commun..

[3] Aupérin A., Le Péchoux C., Rolland E., Curran W.J., Furuse K., Fournel P., Belderbos J., Clamon G., Ulutin H.C., Paulus R. (2010). Meta-analysis of concomitant versus sequential radiochemotherapy in locally advanced non-small-cell lung cancer. J. Clin. Oncol..

[4] Eberhardt W.E.E., De Ruysscher D., Weder W., Le Péchoux C., De Leyn P., Hoffmann H., Westeel V., Stahel R., Felip E., Peters S. (2015). 2nd ESMO Consensus Conference in Lung Cancer: Locally advanced stage III non-small-cell lung cancer. Ann. Oncol..

[5] Daly M.E., Singh N., Ismaila N., Antonoff M.B., Arenberg D.A., Bradley J., David E., Detterbeck F., Früh M., Gubens M.A. (2022). Management of Stage III Non–Small-Cell Lung Cancer: ASCO Guideline. J. Clin. Oncol..

[6] Antonia S.J., Villegas A., Daniel D., Vicente D., Murakami S., Hui R., Yokoi T., Chiappori A., Lee K.H., de Wit M. (2017). Durvalumab after Chemoradiotherapy in Stage III Non-Small-Cell Lung Cancer. N. Engl. J. Med..

[7] Taugner J., Eze C., Käsmann L., Roengvoraphoj O., Gennen K., Karin M., Petrukhnov O., Tufman A., Belka C., Manapov F. (2020). Pattern-of-failure and salvage treatment analysis after chemoradiotherapy for inoperable stage III non-small cell lung cancer. Radiat. Oncol..

[8] Bradley J.D., Hu C., Komaki R.R., Masters G.A., Blumenschein G.R., Schild S.E., Bogart J.A., Forster K.M., Magliocco A.M., Kavadi V.S. (2020). Long-Term Results of NRG Oncology RTOG 0617: Standard- Versus High-Dose Chemoradiotherapy with or without Cetuximab for Unresectable Stage III Non–Small-Cell Lung Cancer. J. Clin. Oncol..

[9] Remon J., Soria J.-C., Peters S. (2021). Early and locally advanced non-small-cell lung cancer: An update of the ESMO Clinical Practice Guidelines focusing on diagnosis, staging, systemic and local therapy. Ann. Oncol..

[10] Singh N., Daly M.E., Ismaila N. (2023). Management of Stage III Non–Small-Cell Lung Cancer: ASCO Guideline Rapid Recommendation Update. J. Clin. Oncol..

[11] Faivre-Finn C., Vicente D., Kurata T., Planchard D., Paz-Ares L., Vansteenkiste J.F., Spigel D.R., Garassino M.C., Reck M., Senan S. (2021). Four-Year Survival with Durvalumab After Chemoradiotherapy in Stage III NSCLC—An Update From the PACIFIC Trial. J. Thorac. Oncol..

[12] Zhou Q., Chen M., Jiang O., Pan Y., Hu D., Lin Q., Wu G., Cui J., Chang J., Cheng Y. (2022). Sugemalimab versus placebo after concurrent or sequential chemoradiotherapy in patients with locally advanced, unresectable, stage III non-small-cell lung cancer in China (GEMSTONE-301): Interim results of a randomised, double-blind, multicentre, phase 3 trial. Lancet Oncol..

[13] Wang Y., Zhang T., Wang J., Zhou Z., Liu W., Xiao Z., Deng L., Feng Q., Wang X., Lv J. (2023). Induction Immune Checkpoint Inhibitors and Chemotherapy Before Definitive Chemoradiation Therapy for Patients with Bulky Unresectable Stage III Non-Small Cell Lung Cancer. Int. J. Radiat. Oncol. Biol. Phys..

[14] Provencio M., Campos B., Guirado M., Vila L., Campelo R.G., Dorta M., Estévez S.V., Ferrández A., Sala M.Á., Ortega A.L. (2025). Induction chemo-immunotherapy followed by chemo-radiotherapy and immunotherapy maintenance in stage III NSCLC (APOLO): A phase 2 trial. Nat. Commun..

[15] Galluzzi L., Humeau J., Buqué A., Zitvogel L., Kroemer G. (2020). Immunostimulation with chemotherapy in the era of immune checkpoint inhibitors. Nat. Rev. Clin. Oncol..

[16] Golden E.B., Apetoh L. (2015). Radiotherapy and immunogenic cell death. Semin. Radiat. Oncol..

[17] Sharabi A.B., Lim M., DeWeese T.L., Drake C.G. (2015). Radiation and checkpoint blockade immunotherapy: Radiosensitisation and potential mechanisms of synergy. Lancet Oncol..

[18] Guan S., Zhang S., Ren K., Li X., Li X., Zhao L. (2023). Induction chemoimmunotherapy may improve outcomes of chemoradiotherapy in patients with unresectable stage III NSCLC. Front. Immunol..

[19] Robins J.M., Hernán M.A., Brumback B. (2000). Marginal structural models and causal inference in epidemiology. Epidemiology.

[20] Bettega F., Mendelson M., Leyrat C., Bailly S. (2024). Use and reporting of inverse-probability-of-treatment weighting for multicategory treatments in medical research: A systematic review. J. Clin. Epidemiol..

[21] Hieke S., Kleber M., König C., Engelhardt M., Schumacher M. (2015). Conditional Survival: A Useful Concept to Provide Information on How Prognosis Evolves over Time. Clin. Cancer Res..

[22] Xing Y., Chang G.J., Hu C., Askew R.L., Ross M.I., Gershenwald J.E., Lee J.E., Mansfield P.F., Lucci A., Cormier J.N. (2010). Conditional survival estimates improve over time for patients with advanced melanoma: Results from a population-based analysis. Cancer.

[23] Royston P., Parmar M.K. (2013). Restricted mean survival time: An alternative to the hazard ratio for the design and analysis of randomized trials with a time-to-event outcome. BMC Med. Res. Methodol..

[24] Anderson J.R., Cain K.C., Gelber R.D. (1983). Analysis of survival by tumor response. J. Clin. Oncol..

[25] Liao J.J.Z., Liu G.F., Wu W.-C. (2020). Dynamic RMST curves for survival analysis in clinical trials. BMC Med. Res. Methodol..

[26] Garassino M.C., Khalifa J., Reck M., Chouaid C., Bischoff H., Reinmuth N., Cove-Smith L., Mansy T., Cortinovis D.L., Migliorino M.R. (2025). Durvalumab after sequential chemoradiotherapy in unresectable stage III non-small-cell lung cancer—Final analysis from the phase II PACIFIC-6 trial. ESMO Open.

[27] Girard N., Bar J., Baas P., Chouaid C., Christoph D.C., Field J.K., Fietkau R., Garassino M.C., Lopez P.G., Gregorc V. (2026). Real-world 5-year outcomes with durvalumab after chemoradiotherapy in unresectable stage III NSCLC. ESMO Open.

[28] Waterhouse D.M., Garon E.B., Chandler J., McCleod M., Hussein M., Jotte R., Horn L., Daniel D.B., Keogh G., Creelan B. (2020). Continuous Versus 1-Year Fixed-Duration Nivolumab in Previously Treated Advanced Non–Small-Cell Lung Cancer: CheckMate 153. J. Clin. Oncol..

[29] Sun L., Bleiberg B., Hwang W.-T., Marmarelis M.E., Langer C.J., Singh A., Cohen R.B., Mamtani R., Aggarwal C. (2023). Association Between Duration of Immunotherapy and Overall Survival in Advanced Non–Small Cell Lung Cancer. JAMA Oncol..

[30] Sun L., Cohen R.B., D’avella C.A., Singh A.P., Schoenfeld J.D., Hanna G.J. (2024). Overall Survival, Treatment Duration, and Rechallenge Outcomes with ICI Therapy for Recurrent or Metastatic HNSCC. JAMA Netw. Open.

[31] Hu Y., Lu T., Zhang H., Chen B., Pan J., Li J., Gong X., Li H., Huang Y., Lu N. (2025). Efficacy and optimal duration of maintenance immunotherapy following systemic chemoimmunotherapy and locoregional radiotherapy in de novo metastatic nasopharyngeal carcinoma: A multicenter retrospective cohort study. Int. J. Cancer.

