# Supplementary material for: Efficacy and Time-Dependent Pattern of Consolidation Immunotherapy in Stage III Non-Small Cell Lung Cancer After Induction Chemoimmunotherapy and Radiotherapy: A Dual-Center Retrospective Cohort Study
Source: Cancers (Basel). 2026 Jun 23;18(13):2035. doi: 10.3390/cancers18132035 (PMC13360170; doi:10.3390/cancers18132035)
Supplement: Supplementary file 1 [file cancers-18-02035-s001.zip › cancers-4338080-supplementary.pdf]

Table S1. Additional baseline characteristics of patients according to consolidation immunotherapy

Variable	Overall cohort		*P* value
	Non-consolidation immunotherapy group (n=105)	Consolidation immunotherapy group (n=65)	
T category			0.44
1	6 (5.7)	7 (10.8)	
2	29 (27.6)	13 (20.0)	
3	27 (25.7)	20 (30.8)	
4	43 (41.0)	25 (38.5)	
N category			0.62
0	4 (3.8)	3 (4.6)	
1	9 (8.6)	4 (6.2)	
2	55 (52.4)	29 (44.6)	
3	37 (35.2)	29 (44.6)	
Induction chemotherapy cycles			0.79
＜3	36 (34.3)	21 (32.3)	
≥3	69 (65.7)	44 (67.7)	
Induction immunotherapy cycles			0.90
＜3	41 (39.0)	26 (40.0)	
≥3	64 (61.0)	39 (60.0)	
Immune checkpoint inhibitors			0.92
Tislelizumab	41 (39.0)	26 (40.0)	
Sintilimab	19 (18.1)	12 (18.5)	
Pembrolizumab	12 (11.4)	11 (16.9)	
Camrelizumab	13 (12.4)	5 (7.6)	
Toripalimab	6 (5.7)	4 (6.2)	
Nivolumab	7 (6.7)	3 (4.6)	
Others	7 (6.7)	4 (6.2)	
Subsequent treatment after disease progression	54*	28*	0.42
Chemotherapy	8 (14.8)	3 (10.7)	
Immunotherapy/ targeted therapy	5 (9.3)	7 (25.0)	

Chemoimmunotherapy/ targeted therapy	22 (40.7)	8 (28.6)
Radiotherapy ±systemic therapy	6 (11.1)	3 (10.7)
Unknown	13 (24.1)	7 (25.0)

* Among the 109 PFS events, 27 were deaths without documented disease progression. Among the remaining 82 patients with confirmed disease progression, 54 belonged to the non-consolidation immunotherapy group and 28 to the consolidation immunotherapy group.

Table S2. Adverse events in consolidation immunotherapy and non-consolidation immunotherapy group.

Event, No. of patients (%)	Consolidation immunotherapy group (n=65)				Non-consolidation immunotherapy group (n=105)				*P* value for all grade	*P* value for Grade 3-4
	Any grade	Grade 1−2	Grade 3−4	Grade 5	Any grade	Grade 1−2	Grade 3−4	Grade 5		
Hematologic toxic effects										
Anemia	54 (83.1)	52 (80.0)	2 (3.1)	0	86 (81.9)	77 (73.3)	9 (8.6)	0	0.85	0.21
Myelosuppression	33 (50.8)	23 (35.4)	10 (15.4)	0	48 (45.7)	34 (32.4)	14 (13.3)	0	0.52	0.71
Blood creatinine increased	9 (13.8)	9 (13.8)	0	0	11 (10.5)	11 (10.5)	0	0	0.51	1
Hypothyroidism	13 (20)	12 (18.5)	1 (1.5)	0	12 (11.4)	8 (7.6)	4 (3.8)	0	0.13	0.65
ALT/AST increased	19 (29.2)	19 (29.2)	0	0	38 (36.2)	35 (33.3)	3 (2.9)	0	0.35	0.29
Hyponatremia	20 (30.8)	20 (30.8)	0	0	41 (39)	40 (38)	1 (1)	0	0.27	1
Hypokalemia	11 (16.9)	10(15.4)	1 (1.5)	0	23 (21.9)	22 (20.9)	1 (1)	0	0.43	1
Hypomagnesaemia	8 (12.3)	8 (12.3)	0	0	17 (16.2)	17 (16.2)	0	0	0.49	1
Hypocalcemia	5 (7.7)	5 (7.7)	0	0	15 (14.3)	15 (14.3)	0	0	0.20	1
Hypoglycemia	2 (3.1)	2 (3.1)	0	0	4 (3.8)	4 (3.8)	0	0	1	1
Hyperglycosemia	39 (60)	36 (55.4)	3 (4.6)	0	58 (55.2)	50 (47.6)	8 (7.6)	0	0.54	0.54
Hypercholesterolemia	37 (56.9)	37 (56.9)	0	0	54 (51.4)	54 (51.4)	0	0	0.49	1
Hyperkalemia	0	0	0	0	1 (1)	1 (1)	0	0	1	1
Hypermagnesemia	2 (3.1)	2 (3.1)	0	0	4 (3.8)	4 (3.8)	0	0	1	1
Hypercalcemia	4 (6.2)	4 (6.2)	0	0	4 (3.8)	4 (3.8)	0	0	0.48	1
Nonhematologic toxic effects										
Pneumonia	53 (81.5)	50 (76.9)	3 (4.6)	0	79 (75.2)	73 (70.4)	5 (4.8)	1	0.34	1
Pyrexia	10 (15.4)	10 (15.4)	0	0	8 (7.6)	8 (7.6)	0	0	0.11	1
Rash	2 (3.1)	2 (3.1)	0	0	4 (3.8)	4 (3.8)	0	0	1	1
Nausea and Vomiting	7 (10.8)	7 (10.8)	0	0	14 (13.3)	14 (13.3)	0	0	0.62	1
Decreased appetite	17 (26.2)	17 (26.2)	0	0	24 (22.9)	24 (22.9)	0	0	0.63	1
Diarrhea	1 (1.5)	1 (1.5)	0	0	3 (2.9)	3 (2.9)	0	0	1	1

Abbreviations: AST, aspartate aminotransferase; ALT, alanine aminotransferase.

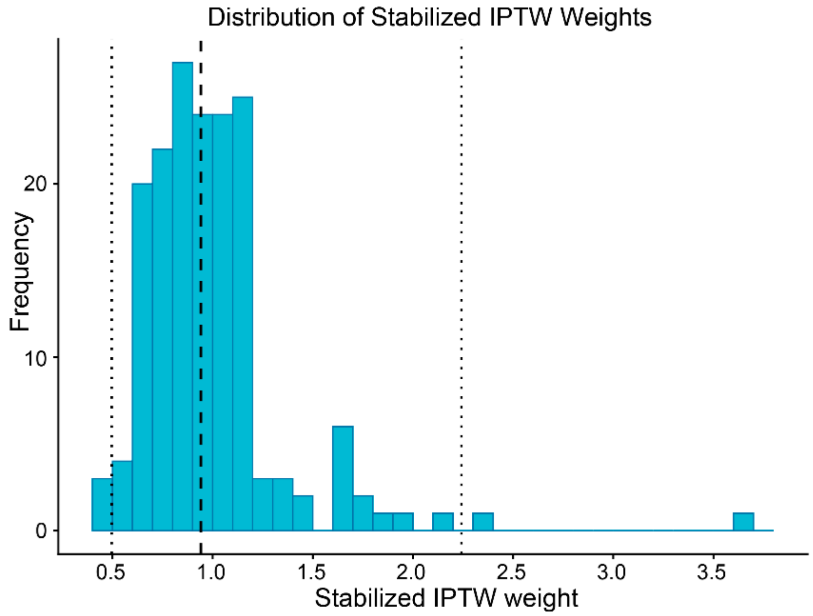

Figure S1. Distribution of stabilized inverse probability of treatment weighting (IPTW) weights. The histogram depicts the distribution of stabilized IPTW weights in the study cohort. The thick dashed vertical line represents the median stabilized IPTW weight, whereas the two thinner dashed vertical lines represent the 1st and 99th percentiles, respectively.

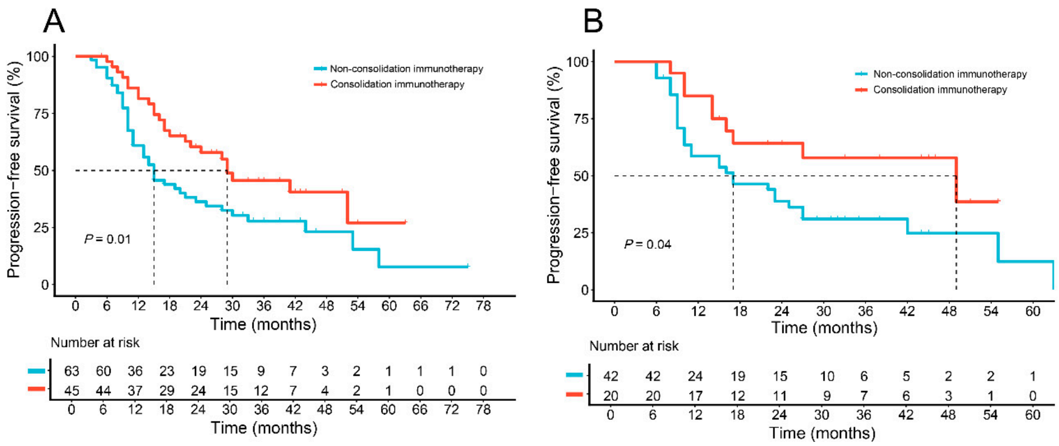

Figure S2. Kaplan–Meier curves of progression-free survival stratified by concurrent chemotherapy status. Progression-free survival was compared between the consolidation immunotherapy and non-consolidation immunotherapy groups among patients who received concurrent chemotherapy (A) and those who did not receive concurrent chemotherapy (B).
